# Early results of Rigo-Chêneau type brace treatment

**DOI:** 10.1186/1748-7161-7-S1-O33

**Published:** 2012-01-27

**Authors:** T Maruyama, Y Nakao, H Yamada

**Affiliations:** 1 Saitama Medical Center, Saitama Medical University, Kawagoe, Japan

## Background

We have been using Rigo-Chêneau type brace for the treatment of idiopathic scoliosis since 2007 [1,2]. Curves other than the upper thoracic main curve were the subjects of the treatment. Most patients wore their brace as part-time, at home or at night.

## Purpose

To evaluate early results of Rigo-Chêneau type brace treatment.

## Materials and methods

A total of 54 patients, 49 females and 5 males, were included in the analysis. Average age at the beginning of the treatment was 12.5 years (10 to 15). Risser sign was 0 in15, I in 9, II in 15, III in 5 and IV in 10 patients. Curve pattern was thoracic (T) in 25, thoracolumbar or lumbar (TL) in 10 and double (D) in 19 patients.

## Results

Average Cobb angle before treatment was 36.5°, which was reduced in the trial brace to 23.2°: correction rate was 36% (34% for T, 69% for TL, and 31% for D curve). Of 54 patients, 20 met the inclusion criteria of the SRS brace study (Risser 0-2, Cobb angle 25-40° ) and six of them reached skeletal maturity during the treatment period. Three of them (50%) progressed more than 6°; however, only one patient progressed more than 10°.

## Conclusions

Although early experience suggested better results than the natural history, accumulation of data will be necessary to determine the effectiveness of the treatment with Rigo-Chêneau type brace.
